# Study on Damage Mechanisms and Blast Mitigation of GFRPU and Coated Steel Plates

**DOI:** 10.3390/polym18202602

**Published:** 2026-10-21

**Authors:** Xiaojun Ye, Quanmin Xie, Dongxian Ye, Rui Zhang, Fuyin Gao, Yanshu Fu, Yongkang Xu, Yaoli Hu, Weibo Huang, Longqiang Liang

**Affiliations:** 1 State Key Laboratory of Precision Blasting, Jianghan University, Wuhan 430056, China; xuyongkang0828@163.com (Y.X.); peakyl99125@163.com (Y.H.); 2 Hubei Key Laboratory of Blasting Engineering, Jianghan University, Wuhan 430056, China; 3 Nanchang Institute of Science and Technology, Nanchang 330108, China; yexiaojun0512@126.com (X.Y.); yedongxian2@163.com (D.Y.); 4 School of Aeronautics Mechanical and Electrical Engineering, Jiangxi Flight University, Nanchang 330014, China; zhangrui@jxfu.edu.cn (R.Z.); yshfu@jxfu.edu.cn (Y.F.); 5 Department of Engineering Technology and Applications, Army Infantry Academy of PLA, Nanchang 330103, China; blastinggaofuyin@163.com; 6 School of Civil Engineering, Qingdao University of Technology, Qingdao 266525, China; spua@163.com; 7 Qingdao Shamu Advanced Material Co., Ltd., Qingdao 266108, China; 15288972807@163.com

**Keywords:** glass fiber-reinforced polyurea, blast mitigation, strain rate effect, hydrogen bonding, contact explosion

## Abstract

Conventional fabrication processes for fiber-reinforced composites are complex and stepwise, limiting their application in blast protection engineering. In this study, KH550 surface-modified glass fiber powder (1.5 wt%) was incorporated into a polyurea matrix, and a homogeneous glass fiber-reinforced polyurea (GFRPU) composite was successfully synthesized via an optimized one-step spray process. The strain-rate-dependent mechanical behavior (0.01–100 s^−1^), fracture mechanisms, molecular structure, and blast mitigation performance of GFRPU were systematically investigated through mechanical testing, SEM, FTIR, 75 g TNT contact explosion tests, and calibrated and partially verified finite element simulations. Results show that the surface-modified fibers form chemical crosslinks with the matrix, shortening the N–H hydrogen bond length from 3.023 Å to 2.983 Å and increasing the hydrogen bonding degree of the urea carbonyl region from 80.0% to 84.1%, thereby enhancing structural ordering and strength. At a strain rate of 100 s^−1^, the tensile strength of GFRPU reaches 50.27 MPa, 26% higher than that of the polyurea matrix. Under contact explosion, a 4 mm back-face GFRPU coating reduced the center-point deflection of the steel plate by 31.6% and suppressed brittle cracking, with interfacial debonding and coating tensile failure as the dominant failure modes. Considering protective efficiency and structural safety, 4 mm is recommended as the optimal coating thickness.

## 1. Introduction

In recent years, secondary damage caused by building fragments generated from explosions has resulted in substantial casualties and property damage [1,2]. Conventional materials, including reinforced concrete and structural steel plates, exhibit inherently limited resistance to blast loading, necessitating the development and integration of advanced material technologies to significantly improve building-level blast resilience [3,4,5].

Glass fiber, a high-performance textile fiber originally developed for industrial applications and subsequently adopted in protective engineering, is characterized by exceptional tensile strength, a high elastic modulus, and outstanding chemical resistance. These attributes have established it as a widely utilized reinforcement material in structural and protective systems [6,7]. A representative application is glass fiber-reinforced polymer (GFRP), a composite formed by embedding continuous glass fibers within a polymer matrix, which has been widely implemented in civil infrastructure, structural retrofitting, and automotive manufacturing [8]. Polyurea, an emerging high-performance coating material, exhibits superior abrasion resistance, corrosion resistance [9], and waterproofing capability [10] compared with conventional materials, and has been extensively applied in industrial corrosion protection and waterproofing. Meanwhile, polyurea demonstrates high tensile strength, exceptional toughness, and outstanding impact resistance. It can be rapidly spray-applied onto structural surfaces to simultaneously enhance load-bearing capacity and ductility without compromising interfacial adhesion to the substrate [11,12]. Existing studies demonstrate that polyurea mitigates blast-induced structural damage by efficiently absorbing blast wave energy via its exceptional deformability, while simultaneously suppressing fragment ejection—thereby reducing secondary injuries to personnel [1,3].

Glass fiber-reinforced polyurea (GFRPU) composites represent a class of materials fabricated by combining glass fibers with a polyurea matrix [13,14,15]. While preserving the inherent characteristics of each constituent, the properties of the individual components become complementary and interrelated, thereby generating a synergistic effect that yields composites with superior overall performance. Currently, the two primary composite processes for GFRPU composites are milled-fiber compounding and interlayer compounding. In milled-fiber compounding, Song et al. [13]. dispersed short glass fibers into a polyurea matrix to produce GFRPU coatings, which were then applied to reinforced concrete beams. This approach eliminates the need for separate fiber-substrate adhesion, thereby streamlining the integration process. Concurrently, the dispersed fibers significantly enhance the tensile strength and fracture resistance of the polyurea matrix. Experimental results reported by Song et al. [13]. confirmed that GFRPU coatings markedly improve both the ultimate load-bearing capacity and flexural ductility of reinforced concrete members. In contrast, interlayer compounding entails the sequential deposition of discrete glass fiber layers and polyurea interlayers. Tian et al. [16]. implemented this strategy using filament winding, positioning polyurea as a compliant interlayer between a metallic shell and wound fiber layers. Explosion tests and finite element analysis revealed that the polyurea interlayer effectively dampens shell vibrations and mitigates impact-induced stress transfer between the metal liner and fiber layers, thereby suppressing liner buckling under dynamic loading.

Existing GFRPU fabrication processes are inherently stepwise: the materials must undergo surface treatment upon completion of the first-stage preparation, which makes the overall process complex and time-consuming. Such stepwise routes are ill-suited to blast protection engineering, where rapid, large-area, in-situ retrofitting is essential. A further limitation lies in the fibers themselves. In existing GFRPU systems, they are generally incorporated without chemical coupling treatment, leaving the influence of fiber surface modification on the molecular architecture of polyurea—particularly the hydrogen-bonding interactions that govern its strength—largely unquantified. Spray-applied polyurea offers a way out. If its processing advantages are fully exploited to integrate glass fibers with the matrix during the reaction process, the fabrication of GFRPU composites can be accomplished in a single step. Fabrication complexity is thus significantly reduced, while the mechanical properties of the polyurea matrix are improved at the same time.

Building upon established blast mitigation polyurea formulations [1,5,12,17], a surface-modified glass fiber powder was incorporated into the polyurea matrix, and the spray-application process, including mixing protocol, nozzle parameters, and curing conditions, was systematically optimized to fabricate a homogeneous, low-porosity GFRPU composite. Fracture behavior was comprehensively characterized by coupling quasi-static and high-strain-rate mechanical testing with in-situ microstructural analysis of damage evolution mechanisms. The effect of glass fiber surface modification on the molecular architecture of polyurea, specifically hydrogen bonding interactions and crosslink density, was quantitatively assessed by Fourier transform infrared spectroscopy (FTIR). Finite element models of bare and GFRPU-coated steel plates were developed under blast loading, calibrated using experimentally derived constitutive data. Subsequent blast experiments calibrated and partially verified model predictions and directly quantified key protective metrics, thereby establishing an integrated experimental–computational framework to guide the rational design of GFRPU composites for blast mitigation.

## 2. Materials and Sample Preparation

The main components of GFRPU coating synthesized in this paper are presented in Table 1.

In this study, 100-mesh glass fiber powder with a filament diameter of 9 μm was employed as the reinforcing fiber. To ensure interfacial bonding between the glass fibers and the polyurea matrix, the glass fiber powder was surface-modified with KH550 silane coupling agent following the method described in Reference [1]. Glass fiber powder was first dispersed in ethanol at a 1:1 (*v*/*v*) ratio and subjected to ultrasonication for 5–10 min to remove the commercial sizing agent from its surface. The treated powder was then washed repeatedly with deionized water and lyophilized. Subsequently, the lyophilized powder was redispersed in an aqueous ethanol solution (ethanol/water, *v*/*v*) and ultrasonicated for 3–5 min to achieve homogeneous dispersion. Then, ammonia was introduced into the solution and further ultra-sonicated for 3–5 min. KH550 was then introduced and allowed to react under continuous stirring for 12–17 h. After reaction completion, the functionalized glass fiber powder was recovered via centrifugation, washed thoroughly with deionized water to remove unreacted reagents, and lyophilized again. The final product was stored under desiccated conditions to prevent moisture-induced hydrolysis of silane bonds.

The role of the KH550 treatment is to build a covalent bridge across the fiber–matrix interface [18]. Upon hydrolysis, the ethoxy groups of KH550 form silanols that condense with the surface Si–OH groups of the glass fiber, producing stable Si–O–Si bonds; the outward-oriented γ-aminopropyl groups then react with the isocyanate groups of Component A during spraying to form urea linkages, thereby tethering the fibers covalently to the polyurea network. This interfacial chemistry promotes load transfer from the compliant matrix to the stiff fibers and strengthens the hydrogen bonding of the surrounding matrix, as quantified in Section 4.2. After surface treatment, the glass fiber powder was freeze-dried to prevent residual moisture from reacting with Component A of the polyurea. To ensure uniform dispersion of the glass fiber powder within the polyurea matrix, the powder was ground after freeze-drying to prevent agglomeration.

GFRPU coating was fabricated using SULZER MixCoat™ DPS 1500-01 (DPS 1500-01, Sulzer Mixpac Ltd., Winterthur, Switzerland) mixing spray system. To enhance coating density and minimize void content, polyurea Components A and B were individually preheated and vacuum-degassed prior to mixing. Specifically, Components A and B were weighed at a mass ratio of 1.1:1, then preheated in an electric oven at 80 °C for 60 min to reduce viscosity and improve flow characteristics. Following preheating, surface-modified glass fiber powder was incorporated into Component B and mechanically stirred to achieve uniform dispersion. The resulting suspension was then subjected to heated ultrasonic treatment (80 °C, 15 min) to break agglomerates and further stabilize fiber dispersion. Subsequently, both components were transferred to a vacuum oven at 80 °C and degassed for 30 min to eliminate entrapped air. After degassing, Components A and B were loaded into the respective material drums of the DPS 1500-01 system, calibrated to deliver a precise 1:1 volumetric mix ratio during spraying. Following spray application, the as-prepared GFRPU was cured for 7 days under ambient conditions of 23 ± 2 °C and 60 ± 15% relative humidity, and was subsequently subjected to mechanical property testing. GFRPU coatings containing 0.5, 1.0, 1.5, and 2.0 wt% modified glass fiber powder were fabricated via the one-step spray process and evaluated under uniaxial tensile loading. Tensile strength increased gradually with fiber content, reaching a maximum at 1.5 wt%. Beyond this content, however, the strength no longer followed a stable trend: at 2.0 wt%, the measured values became scattered and unstable. This loss of stability coincided with a marked rise in the viscosity of Component B, incomplete disruption of fiber agglomerates during ultrasonic dispersion, and the appearance of visible agglomerates—observations consistent with the fiber-content-dependent dispersion limitations reported by Qiao et al. [6]. Higher powder loadings also induced feed instability in the spray system. A fiber content of 1.5 wt% was therefore selected as the optimal balance among mechanical reinforcement, dispersion homogeneity, and spray processability.

## 3. Experiments

### 3.1. Mechanical Performance Test

To investigate the deformation behavior of GFRPU under different strain rates, mechanical property tests were conducted in accordance with ASTM D412 Standard Test Methods for Vulcanized Rubber and Thermoplastic Elastomers. The low-strain-rate mechanical properties were measured using an MZ-4000D1 universal testing machine (Jiangsu Mingzhu Test Machinery Co., Ltd., Yangzhou, China) at strain rates of 0.01 s^−1^ and 0.1 s^−1^. The high-strain-rate mechanical properties of GFRPU were measured using an INSTRON VHS 160-100/20 high-speed hydraulic servo material testing machine (Instron Corporation, Boston, MA, USA) at strain rates of approximately 1 s^−1^, 10 s^−1^, and 100 s^−1^. For each material and each strain rate (0.01, 0.1, 1, 10, and 100 s^−1^), five dumbbell-shaped specimens were tested; only tests exhibiting fracture strictly within the gauge length were deemed valid. Unless otherwise specified, the stress–strain curves presented herein are representative traces selected based on minimal deviation from the arithmetic mean tensile strength calculated from the five valid replicates.

To investigate the fracture mechanisms of GFRPU, tensile fracture surfaces were examined using a JSM 6700F scanning electron microscope (SEM) (JEOL Ltd., Tokyo, Japan)following mechanical testing, so as to analyze the distribution and failure modes of glass fibers in the composite as well as the interfacial bonding between the fibers and the matrix. The influence of glass fibers on the fracture morphology of the polyurea matrix was also investigated.

### 3.2. FTIR

To investigate the influence of modified glass fiber powder on the molecular structure of polyurea, FTIR analysis was performed on the polyurea matrix and GFRPU using VERTEX 70 FTIR spectrometer (Bruker Corporation, Karlsruhe, Germany), with particular attention to the absorption spectra in the amine. Prior to FTIR analysis, the specimen surfaces were cleaned with anhydrous ethanol. The FTIR measurements were performed with 32 scans at a resolution of 4 cm^−1^ over the spectral region of 4000–500 cm^−1^.

### 3.3. Explosion Test

To investigate the fracture characteristics of GFRPU under blast loading and its blast mitigation performance for metallic structures, GFRPU was spray-applied onto the back blast face of steel plates. Contact explosion tests were subsequently conducted on unprotected steel plates and GFRPU-coated steel plates using 75 g TNT charges. Q235A steel plates with dimensions of 300 mm × 300 mm × 8 mm were used as the substrate. Mounting holes were pre-drilled around the plate perimeter to facilitate rapid fixation of the steel plate during testing.

**Figure 1:**
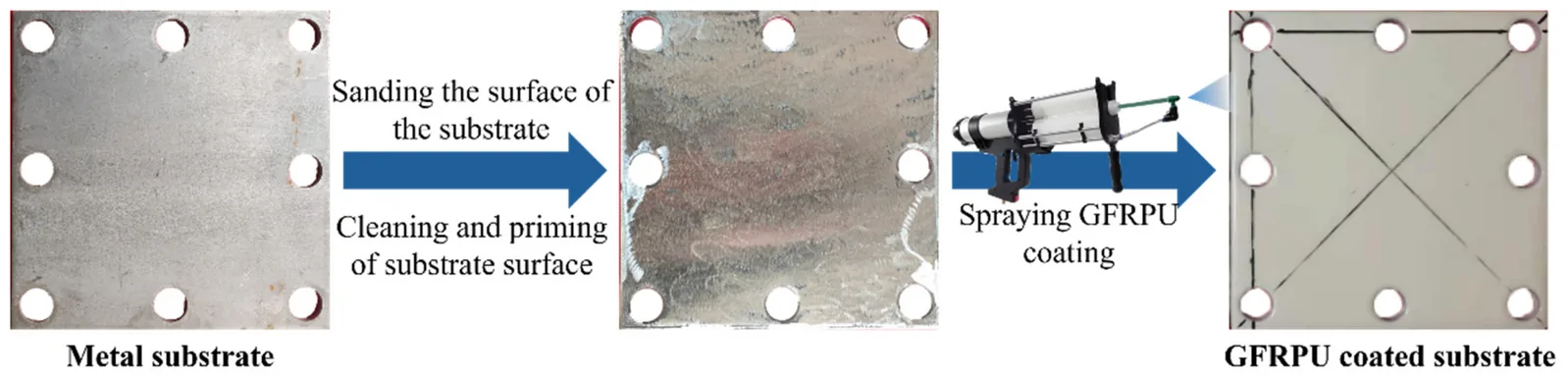
GFRPU-coated steel plate lamination process.

Prior to coating application, the steel plate surface was sanded and sprayed with an epoxy-modified polyurethane primer to enhance adhesion between the coating and the steel substrate. A 4 mm GFRPU coating was spray-applied onto the back blast face of the steel plate using the DPS 1500-01, with the composite fabrication process illustrated in Figure 1.

**Figure 2:**
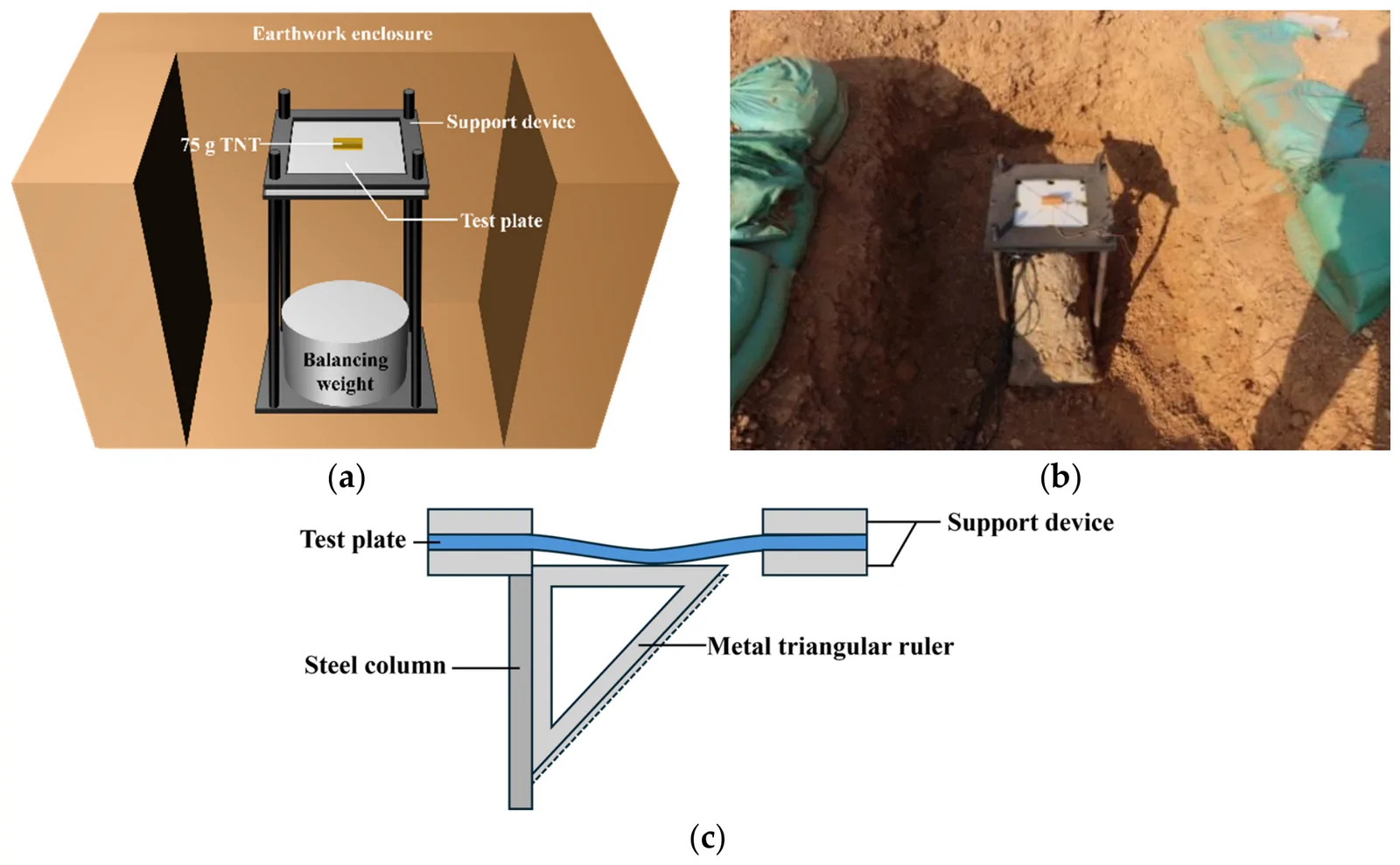
Explosion test device setup: (**a**) schematic diagram of the test support; (**b**) practical test setup; (**c**) measuring device.

Contact explosion tests were performed to compare unprotected steel plates with back blast face coated steel plates, with the specimen support and loading configuration illustrated in Figure 2. The blast test frame was installed in an earthen pit, with counterweights strategically configured at its base to provide overturning resistance and ensure structural stability under blast loading. The specimen was rigidly clamped within the frame using a steel restraining plate featuring a centrally located circular cutout, and a 75 g cylindrical TNT charge was positioned concentrically with the geometric center of the specimen surface.

After each blast test, the post-blast deformation of the specimen was quantified as follows. The peripheral region of the steel plate clamped beneath the restraining plate remained essentially undeformed during the explosion and was therefore used to define the reference plane, which coincides with the undeformed plane of the plate. The center-point deflection was measured as the perpendicular distance from the center of the back-blast face of the plate to this reference plane using a measuring device (Figure 2c), each plate was measured three times at the center point. Accordingly, the center-point deflection reported in this work denotes the total maximum permanent displacement of the plate center relative to the initial flat configuration, in which the overall bending of the plate is inherently included.

It should be noted that one specimen was tested for each configuration in this preliminary comparative study. All tests were performed under strictly identical charge mass, geometry, and clamping conditions to ensure internal consistency of the comparison.

## 4. Experimental Results and Analysis

### 4.1. Mechanical Properties Under Different Strain Rates

Existing studies consistently demonstrate that polyurea exhibits a pronounced strain-rate sensitivity, characterized by distinct mechanical responses across varying strain-rate ranges [1,5,17]. The incorporation of modified glass fiber powder induces microstructural heterogeneity in polyurea and modifies its molecular architecture, yielding a more complex hierarchical structure in GFRPU, thereby altering its strain-rate sensitivity. Consequently, quantifying the strain-rate-dependent mechanical response of GFRPU is critical for designing blast mitigation structural systems.

**Figure 3:**
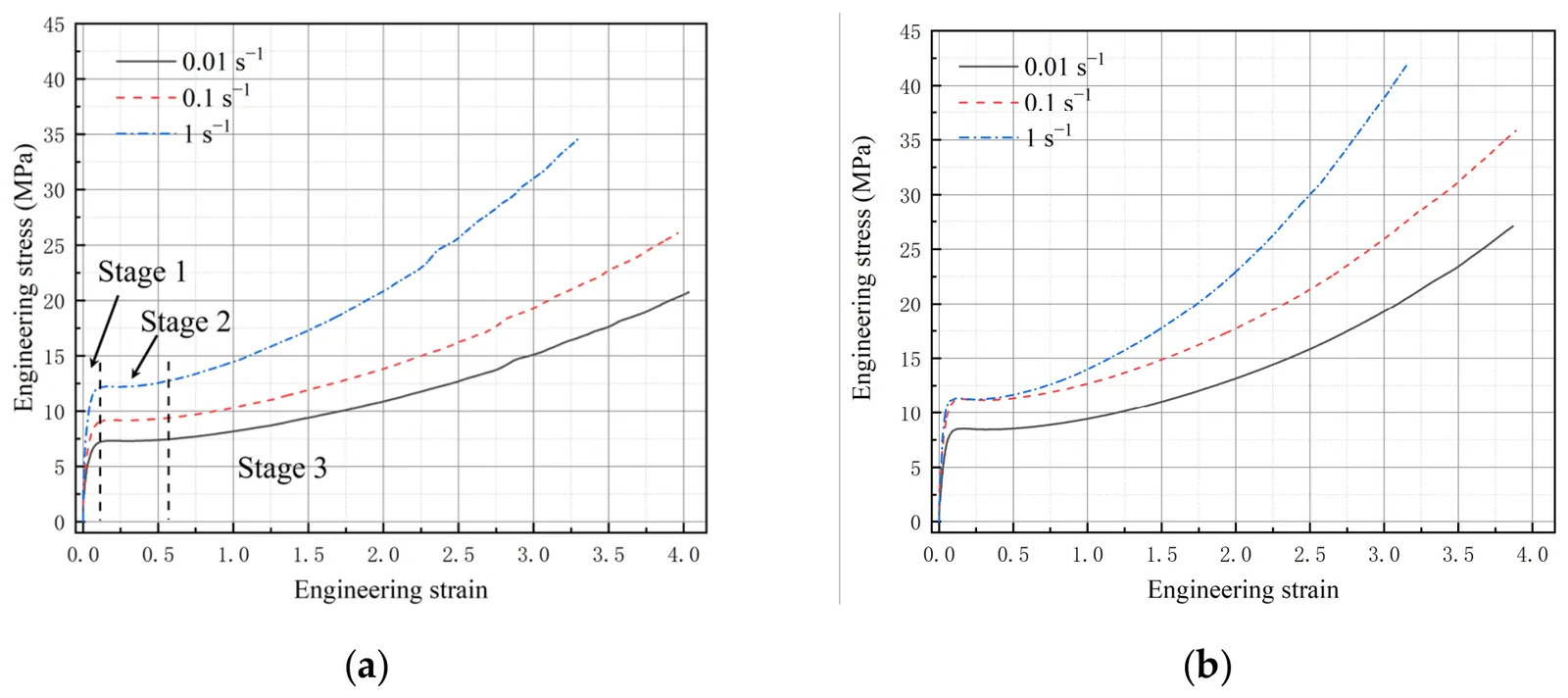
Engineering stress-engineering strain curves of polyurea at low strain rates; (**a**) Shamu T4; (**b**) GFRPU.

First, the quasi-static mechanical response of polyurea was characterized at strain rates of 0.01 s^−1^, 0.1 s^−1^, and 1 s^−1^. The corresponding engineering stress–engineering strain curves for Shamu T4 and GFRPU are shown in Figure 3.

It can be seen from Figure 3 that polyurea exhibits typical elastomeric tensile behavior at all tested strain rates, with pronounced strain rate sensitivity. The engineering stress–engineering strain curves of polyurea with and without modified glass fiber addition display similar mechanical characteristics. Taking the matrix polyurea (Figure 3a) as an example, the deformation response can be broadly divided into three stages: the linear elastic deformation stage (Stage 1), the hyperelastic stage (Stage 2), and the nonlinear deformation stage (Stage 3). During the linear elastic deformation stage, the tensile behavior of polyurea essentially conforms to Hooke’s law, with stress and strain exhibiting a linear relationship over a strain range of approximately 0–0.05. The fitted elastic moduli of polyurea at strain rates of 0.01 s^−1^, 0.1 s^−1^, and 1 s^−1^ are 77.64 ± 3.26 MPa, 96.78 ± 2.32 MPa, and 130.85 ± 2.75 MPa, respectively, indicating a pronounced increase with strain rate. For GFRPU, the elastic moduli at the corresponding strain rates are 90.35 ± 3.61 MPa, 111.21 ± 4.89 MPa, and 119.47 ± 4.90 MPa, respectively. Compared with the matrix polyurea, the increase in elastic modulus is relatively moderate. The elastic behavior of polyurea in the linear elastic deformation stage arises from small deformations induced by segmental and bond-angle motions of the molecular chains under tension. Upon incorporation of modified glass fiber powder, the amino groups attached to the fiber surface react with the isocyanate groups in Component A to form covalent interfacial linkages, thereby restricting intermolecular segmental motion and consequently reducing the strain rate sensitivity in the linear elastic deformation stage.

In the hyperelastic stage (Stage 2), the strain of polyurea ranges from approximately 0.05 to 0.6. During this stage, the yield point in the engineering stress–engineering strain response is indistinct, and the stress remains nearly constant. The strain rate effect of polyurea is most pronounced in the nonlinear deformation stage (Stage 3). As the strain rate increases, the tensile curve shifts upward, corresponding to elevated stress. At the microscopic level, the molecular chains of polyurea extend along the tensile direction during this stage, and the increased resistance to segmental motion leads to further stress growth until material fracture. A comparison between matrix polyurea and GFRPU indicates that when the strain rate is increased from 0.01 s^−1^ to 1 s^−1^, the maximum tensile stress of the two materials increases by 67% and 55%, respectively, with the strain rate effect being more pronounced in matrix polyurea. The elastic modulus in the linear elastic deformation stage follows the same trend; the addition of modified glass fiber powder reduces the strain rate sensitivity of matrix polyurea at low strain rates. Moreover, when the strain rate is increased from 0.01 s^−1^ to 1 s^−1^, the elongation at break of both materials decreases by approximately 18% at low strain rates. Quantitatively, the elastic modulus of neat polyurea increases from 77.64 MPa at 0.01 s^−1^ to 130.85 MPa at 1 s^−1^ (a factor of 1.69 over two decades of strain rate), whereas that of GFRPU increases from 90.35 MPa to 119.47 MPa (a factor of 1.32); the rate-sensitivity exponents over this range were quantified by fitting the modulus data to the power-law relation given in Equation (1):

(1) $$E=E_{0}{\left(\frac{\dot{\varepsilon }}{{\dot{\varepsilon }}_{0}}\right)}^{m}$$

where E is the elastic modulus, MPa; $\dot{\varepsilon }$ is the strain rate, s^−1^; $\dot{\varepsilon }$ _0_ is the reference strain rate (0.01 s^−1^); and m = Δlog E/Δlog $\dot{\varepsilon }$ ; is the rate-sensitivity exponent, defined by analogy with the strain-rate sensitivity index m = ∂lnσ/∂ln $\dot{\varepsilon }$ established for polyurea [19]. The fitting yields m = 0.113 for neat polyurea and 0.061 for GFRPU, confirming that the fiber–matrix crosslinks restrict segmental motion and weaken the low-rate sensitivity of the composite.

**Figure 4:**
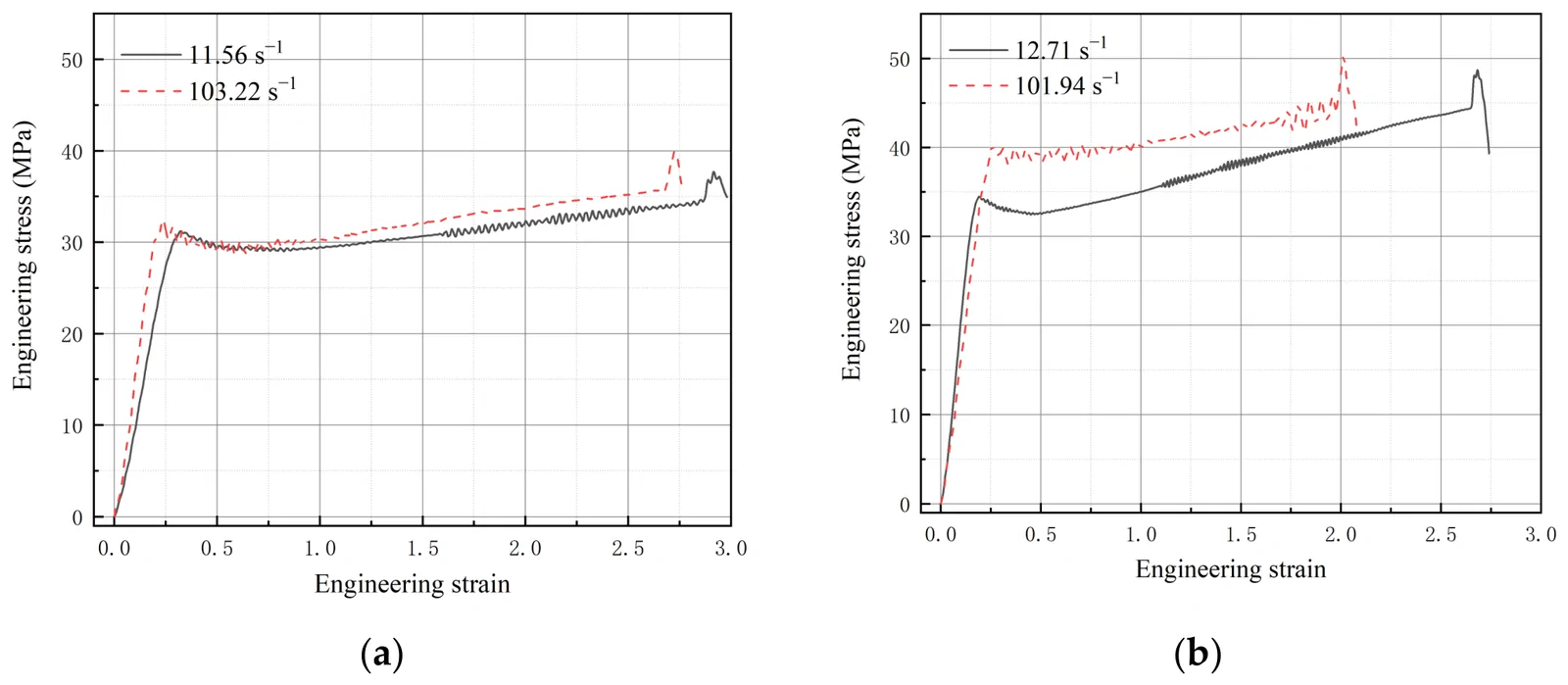
Engineering stress-engineering strain curves of polyurea at medium strain rates: (**a**) Shamu T4; (**b**) GFRPU.

Polyurea exhibits fundamentally distinct deformation mechanisms under low versus intermediate-to-high strain-rate loading. Experimental evidence suggests that polyurea exhibits rubber-like behavior at low strain rates but demonstrates glassy behavior at intermediate-to-high strain rates [1,17,20]. The engineering stress–engineering strain curves of matrix polyurea and GFRPU at intermediate strain rates are presented in Figure 4. Compared with those at low strain rates, the curves of both materials exhibit pronounced yield points at intermediate strain rates. The yield stresses of polyurea matrix and GFRPU at a strain rate of 100 s^−1^ are 32.36 ± 0.87 MPa and 40.15 ± 1.24 MPa, respectively. Regarding elastic modulus, both materials also demonstrate certain improvements. The elastic moduli at 100 s^−1^ are 158.79 MPa and 178.78 MPa, respectively, corresponding to increases of 21% and 50% relative to those at 1 s^−1^. For mechanical strength, the tensile strengths of neat polyurea and GFRPU at 100 s^−1^ are 39.96 ± 1.32 MPa and 50.27 ± 1.86 MPa, respectively, representing substantial enhancements compared with those at low strain rates. A comparison between neat polyurea and GFRPU reveals that, within the intermediate strain rate range, the increases in both elastic modulus and stress of GFRPU with increasing strain rate exceed those of the matrix material, indicating that the composite exhibits more pronounced strain rate sensitivity at high strain rates and that the reinforcing effect of modified glass fiber powder on the matrix material is more significant at intermediate strain rates. This further demonstrates that the composite possesses different deformation mechanisms at low and high strain rates, consistent with the conclusions of Sarvr [21] and Yi [22].

**Figure 5:**
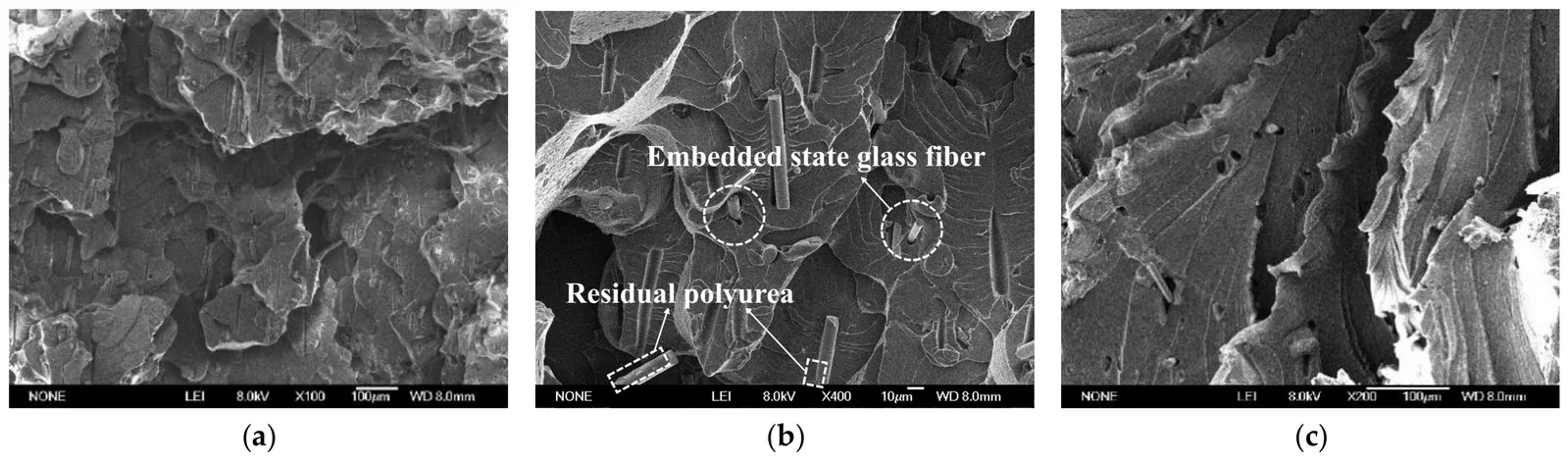
GFRPU coating tensile section characterization areas: (**a**) tensile section; (**b**) partial enlargement of tensile section; (**c**) edge region of tensile section.

To further investigate the fracture failure characteristics of GFRPU, samples were taken from the fracture surfaces of fractured specimens and examined by SEM to observe the microscopic morphology and fracture characteristics, as shown in Figure 5.

Macroscopically, the tensile fracture surface (Figure 5a) exhibits a distinct irregular step-like morphology, with no evident large-area flat regions. This indicates that the fracture surface of GFRPU is not microscopically uniform upon tensile failure, which is attributed to the spray-applied synthesis process of GFRPU and the presence of internal glass fibers. The spray-applied synthesis process introduces a certain amount of air voids within the matrix, resulting in non-uniform stress distribution in the polyurea under tension. Meanwhile, pronounced stress concentration is induced around the modified glass fibers due to their reinforcing effect in GFRPU, thereby producing the step-like damage morphology upon fracture failure. A magnified view of the tensile fracture surface (Figure 5b) reveals that glass fibers and fiber cavities are uniformly distributed across the surface. This indicates that, upon fracture failure of GFRPU, some fibers were ejected under stress and left behind cavities, while other fibers remained adhered to the coating surface after pull-out due to the favorable adhesion between the modified glass fibers and the matrix.

Additionally, a fraction of the fibers remained embedded in the polyurea matrix on the tensile fracture surface (Figure 5b), providing direct evidence of favorable interfacial adhesion between the modified glass fibers and the matrix. At the edge region of the fracture surface (Figure 5c), tearing-dominated damage was observed as a wavy morphology, which arises from the non-uniform stress distribution in this region. Only a limited number of pull-out cavities and few embedded fibers were found there, indicating that the reinforcing fibers are prone to fracture under tearing-dominated damage. Two further morphological features corroborate the strong fiber–matrix bonding. First, the pull-out cavities in Figure 5b are shallow and short, implying that fiber pull-out is strongly resisted and that failure occurs partly within the matrix surrounding the fibers rather than along a weak interface. Second, thin layers of residual polyurea adhere to the surfaces of the pulled-out fibers, as indicated by Figure 5b. Fracture therefore propagated cohesively through the matrix adjacent to the fibers—a direct morphological signature of the effective interfacial adhesion imparted by the KH550 coupling layer.

### 4.2. Effect of Modified Glass Fibers on the Molecular Structure of Polyurea

**Figure 6:**
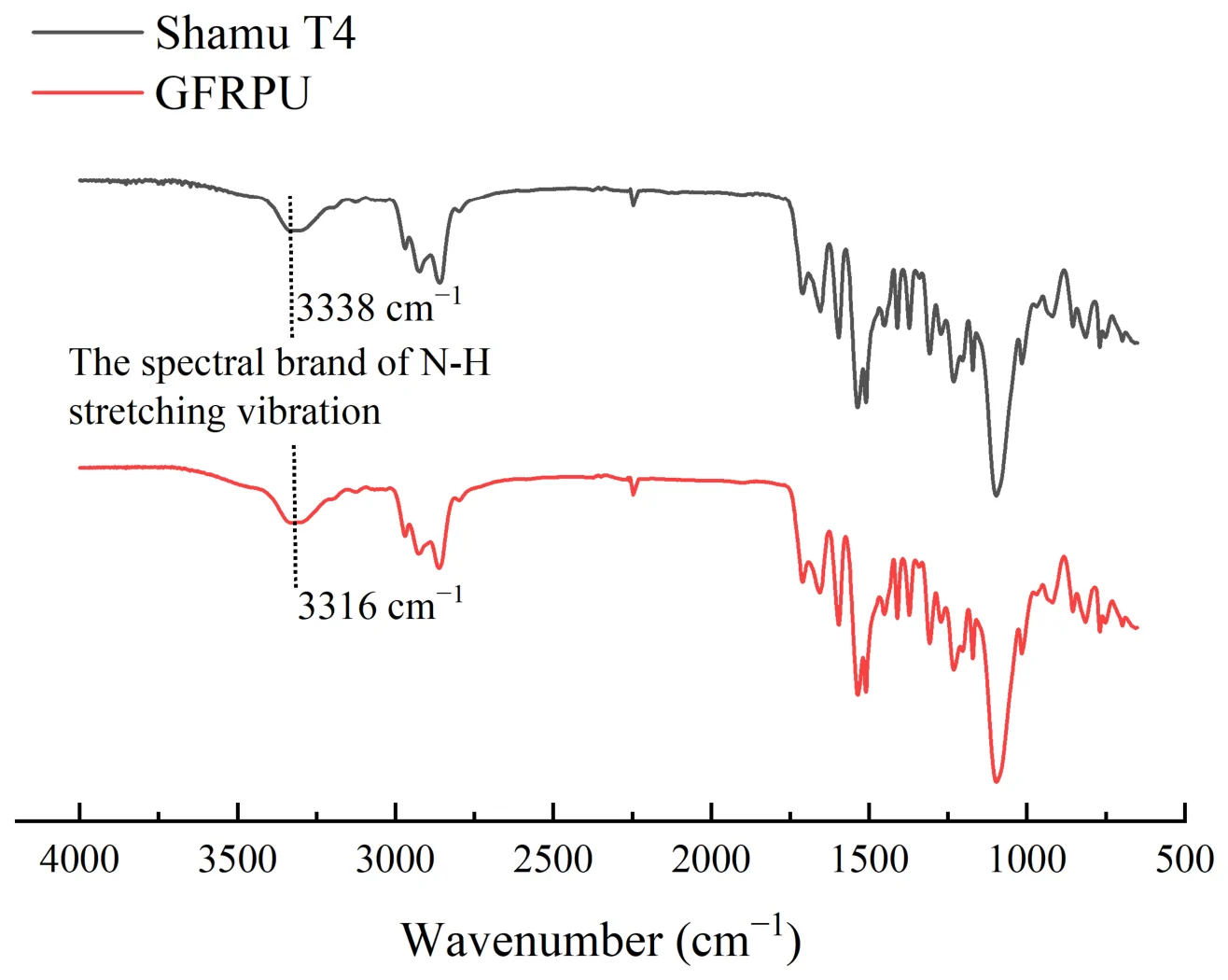
FTIR spectra of Shamu T4 and GFRPU.

To investigate the influence of modified glass fibers on the molecular structure of polyurea, FTIR spectroscopy was employed in this study to compare the characteristic peaks of functional groups in the molecular chains of neat polyurea and GFRPU, with particular emphasis on the effects of modified glass fibers on hydrogen bond length and the degree of hydrogen bonding in polyurea. The FTIR spectra of neat polyurea and GFRPU are presented in Figure 6.

The stretching vibration absorption bands of the characteristic functional groups in the polyurea matrix and GFRPU are generally similar. The peak near 3340 cm^−1^ corresponds to the N–H stretching vibration of amine groups, and the band at 1580–1720 cm^−1^ is attributed to urea carbonyl absorption. These observations indicate that the –NHCONH– structure is present in the molecular architecture of both materials. Additionally, the absorption bands of CH_2_ and CH_3_ side groups appear at 2814–3005 cm^−1^, the C–N absorption band at 1504–1540 cm^−1^, and the C–O–C absorption band at 1047–1126 cm^−1^. Hydrogen bonding is a structural characteristic that directly influences the properties of polyurea [18]. As shown in Figure 6, both materials exhibit characteristic peaks of hydrogen-bonded N–H stretching vibrations near 3340 cm^−1^, whereas the characteristic peak of free N–H stretching vibrations at 3440 cm^−1^ is almost negligible, indicating that the N–H groups in both materials are almost completely hydrogen-bonded. The hydrogen-bonded N–H stretching vibrations of neat polyurea and GFRPU are located at 3338 cm^−1^ and 3316 cm^−1^, respectively, with absorption band frequency shifts (Δν) of 102 cm^−1^ and 124 cm^−1^, respectively. It should be emphasized that FTIR directly probes hydrogen bonding interactions, whereas the formation of covalent urea linkages between the fiber surface and the matrix cannot be resolved spectrally, because the newly formed interfacial urea carbonyl groups overlap with the intrinsic urea carbonyl bands of the matrix. The presence of such covalent linkages is therefore inferred from the established reaction chemistry of KH550—whose terminal –NH_2_ groups react with the –NCO groups of Component A to form urea bonds [18]—and is corroborated by the cohesive matrix failure around pulled-out fibers observed by SEM (Section 4.1) and by the restricted segmental mobility reflected in the reduced low-strain-rate sensitivity of the elastic modulus (Section 4.1). Accordingly, the FTIR results are interpreted here as evidence of enhanced hydrogen bonding and structural ordering, rather than as direct proof of chemical crosslinking. The hydrogen bond length of polyurea is calculated using Equation (2):

(2) $$R=3.21-\frac{\Delta V}{0.548\times 10^{3}}$$

where R denotes the hydrogen bond length, Å; Δν denotes the distance between the non-hydrogen-bonded N–H peak and the hydrogen-bonded N–H peak, cm^−1^. The calculated N–H hydrogen bond length of neat polyurea is 3.023 Å; by comparison, that of GFRPU is 2.983 Å, indicating that the N–H hydrogen bonding is enhanced upon the addition of modified glass fiber powder. Self-deconvolution was performed on the urea carbonyl region (1580–1720 cm^−1^) to investigate the degree of hydrogen bonding in the carbonyl region, and the results are presented in Table 2. The self-deconvolution of the urea carbonyl region and the calculation of the hydrogen-bonding degree follow the method established in our previous work [5], in which the carbonyl band is resolved into ordered hydrogen-bonded, disordered hydrogen-bonded and free sub-bands, and the percentages X_o_, X_diso_ and X_b_ (X_b_ = X_o_ + X_diso_) are obtained from the integrated peak-area ratios.

The total hydrogen bonding degree of the urea carbonyl region in neat polyurea is 80.0%, and the ordered hydrogen-bonded urea carbonyl content is 56.8%. Both the total hydrogen bonding degree and the ordered hydrogen-bonded urea carbonyl content of GFRPU are increased, reaching 84.1% and 59.2%, respectively, indicating that GFRPU possesses the highest degree of structural ordering. The aforementioned hydrogen bonding investigations of the N–H and urea carbonyl regions in neat polyurea and GFRPU demonstrate that, with the incorporation of modified glass fiber powder, the N–H hydrogen bond length decreases, the hydrogen bonding degree of the urea carbonyl increases, and the structural ordering of GFRPU is enhanced, which is macroscopically manifested as an improvement in the mechanical strength of the material. The correlation between these molecular-scale changes and the macroscopic mechanical improvement can be rationalized within the established hydrogen-bonding–property framework of polyurea [10]. The covalently tethered fibers introduce additional hydrogen-bonding sites and constrain the surrounding hard domains, thereby strengthening the average N–H⋯O hydrogen bond and increasing the fraction of ordered hydrogen-bonded carbonyls. The resulting denser and more ordered hydrogen-bond network functions as additional physical crosslinks that restrict segmental motion; macroscopically, this manifests as the increased elastic modulus, yield stress and tensile strength, as well as the reduced low-strain-rate sensitivity of the modulus reported in Section 4.1. The FTIR evidence and the mechanical data are thus consistent with a single underlying mechanism of fiber-induced structural ordering.

It should be emphasized that FTIR directly probes hydrogen bonding interactions, whereas the formation of covalent urea linkages between the fiber surface and the matrix cannot be resolved spectrally, because the newly formed interfacial urea carbonyl groups overlap with the intrinsic urea carbonyl bands of the matrix. The presence of such covalent linkages is therefore inferred from the established reaction chemistry of KH550—whose terminal –NH_2_ groups react with the –NCO groups of Component A to form urea bonds [18]—and is corroborated by the cohesive matrix failure around pulled-out fibers observed by SEM (Section 4.1) and by the restricted segmental mobility reflected in the reduced low-strain-rate sensitivity of the elastic modulus (Section 4.1). Accordingly, the FTIR results are interpreted here as evidence of enhanced hydrogen bonding and structural ordering, rather than as direct proof of chemical crosslinking.

### 4.3. Failure Characteristics of GFRPU-Coated Steel Plate

**Figure 7:**
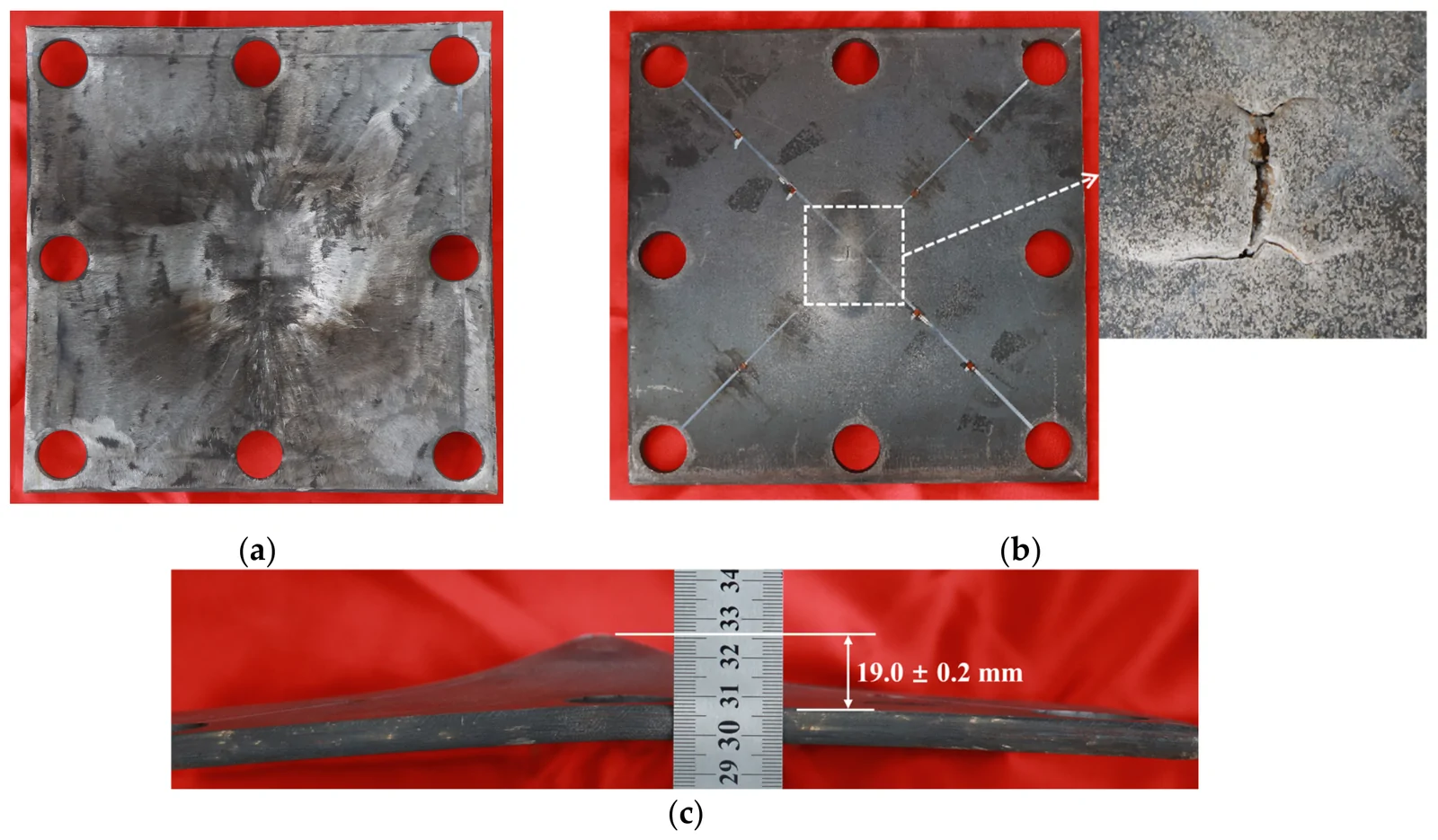
Macroscopic damage morphology of steel plate without coating protection: (**a**) blast face of uncoated steel plate; (**b**) back-blast face of uncoated steel plate; (**c**) side face of uncoated steel plate.

**Figure 8:**
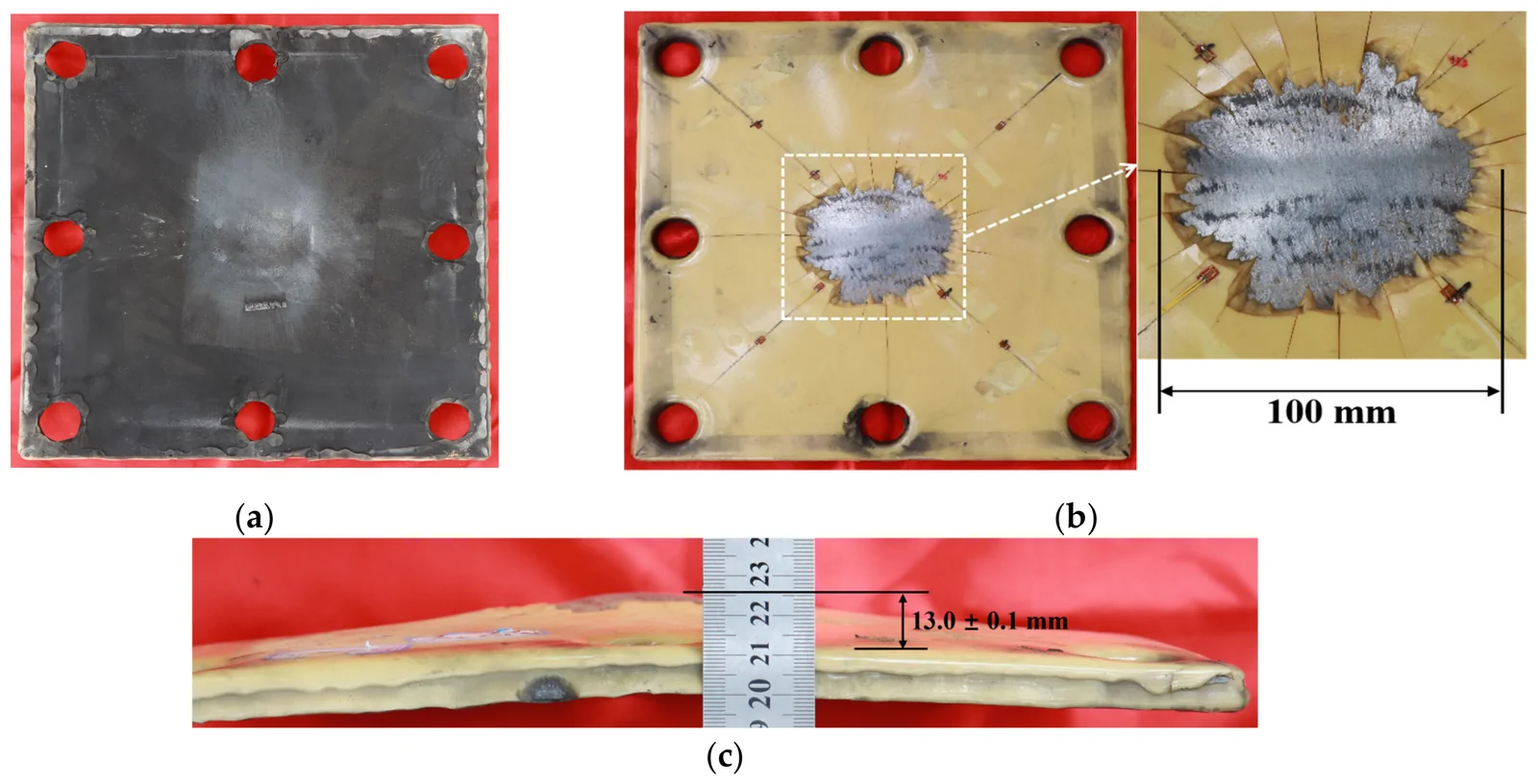
Macroscopic damage morphology of steel plate with coating protection: (**a**) blast face of coated steel plate; (**b**) back-blast face of coated steel plate; (**c**) side face of coated steel plate.

To systematically evaluate the damage mitigation capability and blast mitigation performance of GFRPU, contact explosion tests were performed using 75 g cylindrical TNT charges on both bare steel plates and steel plates retrofitted with a 4 mm-thick GFRPU coating. The test results are shown in Figure 7 and Figure 8.

As shown in Figure 7a, under 75 g TNT contact loading, the front face of the unprotected steel plate exhibited a pit, with minor cracking generated around the pit. The back blast face of the steel plate developed a bulge resembling the shape of the charge (Figure 7b), and a crack formed near the center of the back blast face, indicating that the steel plate was in a state of critical failure. The unprotected steel plate underwent substantial deformation under blast loading, with the center-point deflection measured at approximately 19 ± 0.2 mm, as shown in Figure 7c. The front-face damage of the GFRPU-protected steel plate was essentially similar to that of the unprotected steel plate, with significant deformation occurring in the charge region. However, the macroscopic damage on the back blast face of the GFRPU-protected steel plate was markedly less severe.

From the perspective of back blast face damage, the 75 g TNT contact explosion induced coating debonding in the central region of the back face, producing a debonded zone approximately 100 mm in diameter on the back blast face (Figure 8b), with the coating exhibiting pronounced shear failure. However, examination of the steel plate morphology within the debonded zone reveals that the steel plate remained uncracked, indicating that the polyurea effectively suppressed brittle cracking of the steel plate. From the macroscopic deformation observed on the specimen side (Figure 8c), the center-point deflection of the GFRPU-coated steel plate was 13 ± 0.1 mm, representing a reduction of approximately 31.58% compared with the unprotected steel plate. Evaluating the blast resistance of the structure in terms of plastic deformation under blast loading, the above test results indicate that GFRPU coating protection enhances the blast mitigation of metallic structures subjected to contact explosion.

Based on the macroscopic damage observed in the two specimens after explosion, the impulse transmitted to the steel plate is greatest at the contact interface under contact explosion loading, causing deformation of the steel plate and resulting in pit formation on the front face. Meanwhile, blast loading is characterized by high pressure and high strain rates. During this dynamic process, the steel plate undergoes high-frequency vibration, and a significant velocity gradient develops between the pit vicinity and the unloaded region. Consequently, the pit area is continuously subjected to tensile action from the shock wave, ultimately leading to crack formation at the pit edge, as shown in Figure 7a. When the blast wave propagates to the back blast face of the steel plate, the combined action of the shock wave and the tensile deformation of the back face produces the crack shown in Figure 7b. For the GFRPU-protected steel plate, the interfacial adhesion between GFRPU and the steel plate is markedly weaker than the tensile wave effect. Meanwhile, under the influence of high-frequency vibration generated by the explosion, the GFRPU tends toward a glassy state, and its fracture toughness decreases at the boundary of the large-deformation region of the steel plate. Under the combined action of these two factors, overall debonding of the GFRPU coating occurred on the back face. It should be noted that, owing to the reinforcing effect of the modified glass fiber powder on the polyurea matrix and the discrete distribution of the reinforcement, internal coating fracture is influenced by the fibers, resulting in an oblique fracture morphology in the boundary region of the debonded zone (Figure 8b) rather than a fracture perpendicular to the coating surface.

**Figure 9:**
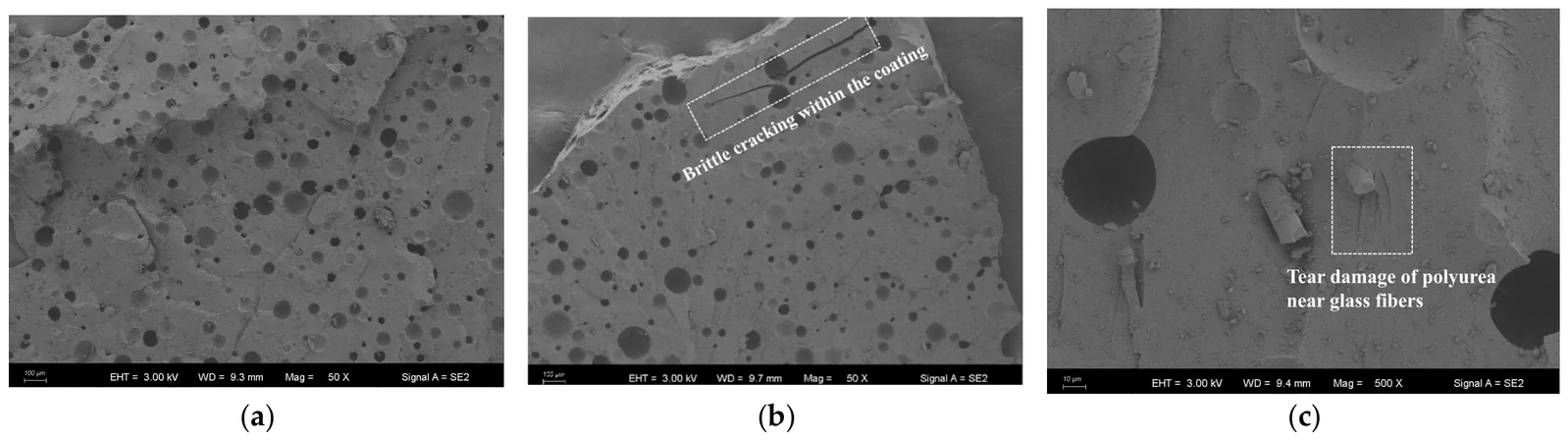
GFRPU coating microfracture morphology: (**a**) brittle fracture of coating section; (**b**) brittle cracking within the coating; (**c**) glass fiber inside the coating.

To further investigate the fracture characteristics of the back blast face coating, samples were taken from the coating fracture surface and examined by scanning electron microscopy to directly reveal the microscopic fracture features; the fracture surface morphology is shown in Figure 9.

The microscopic morphology of the back-face GFRPU fracture surface primarily exhibits three characteristics: large flat fracture regions across the overall fracture surface (Figure 9a), brittle fracture within the coating interior (Figure 9b), and the presence of a small number of embedded fibers (Figure 9c). These flat fracture regions constitute a direct manifestation of brittle fracture in the coating and differ considerably from the step-like planes of the fracture surfaces produced under mechanical loading (Figure 5a), being predominantly characterized by large-area flat regions. This phenomenon is attributed, on the one hand, to the high loading rate of blast loading, which induces brittle fracture, and on the other hand, to the transition of GFRPU into a glassy state resulting from the elevated loading rate.

The brittle fracture within the coating is likewise influenced by the glass transition of polyurea. Meanwhile, this fracture characteristic occurs predominantly at the coating edge, where the impedance mismatch of GFRPU is more severe, tensile action is more readily induced, and brittle fracture is thus more prone to occur. It should be noted that this tensile action also affects the modified glass fibers within the coating. Upon fracture failure, the majority of the internal glass fibers were detached, with only a small fraction remaining embedded in the coating, thereby constituting the third morphological characteristic (Figure 9c). This phenomenon also directly confirms the favorable adhesion between the modified glass fibers and the polyurea matrix, meeting the requirements for composite materials. It is noteworthy that pronounced tearing damage traces were observed in the vicinity of the glass fibers, indicating that the glass fibers altered the stress distribution in the surrounding polyurea matrix. Stress concentration and a reduced strain rate in the vicinity of the glass fibers suppressed brittle fracture in the polyurea matrix. The tearing ridges concentrated around the embedded fibers further indicate that crack propagation was deflected by the fibers, which is only possible when the stress is efficiently transferred across a well-bonded interface.

Based on the above macroscopic and microscopic observations, the failure sequence of the bare and GFRPU-coated steel plates under contact explosion can be summarized in four steps. (i) Localized pitting: the impulse is maximal at the blast face, producing the front-face pit and minor radial cracking common to both configurations (Figure 7a and Figure 8a). (ii) Back blast face tensile cracking of the bare plate: the compressive shock reflects at the back blast face as a tensile wave, which, superimposed on the global bending tension and high-frequency vibration, produces the back blast face bulge and the near-critical central crack (Figure 7b). (iii) Interfacial debonding of the coated plate: the impedance mismatch at the steel–GFRPU interface generates a reflected tensile wave whose amplitude exceeds the interfacial adhesion strength, thereby triggering coating debonding over a circular region approximately 100 mm in diameter, while the substrate remains intact without cracking (Figure 8b). (iv) Brittle coating fracture: at blast loading rates, the polyurea approaches the glassy state; its fracture toughness decreases at the boundary of the large-deformation region, and the coating fails in a brittle, oblique mode whose crack path is deflected by the embedded fibers (Figure 8b and Figure 9), which distinguishes it from the fracture perpendicular to the coating surface that would be expected for neat polyurea.

## 5. Deformation Behavior of GFRPU Coated Steel Plates with Different Thicknesses

### 5.1. Establishment of Simulation Model

**Figure 10:**
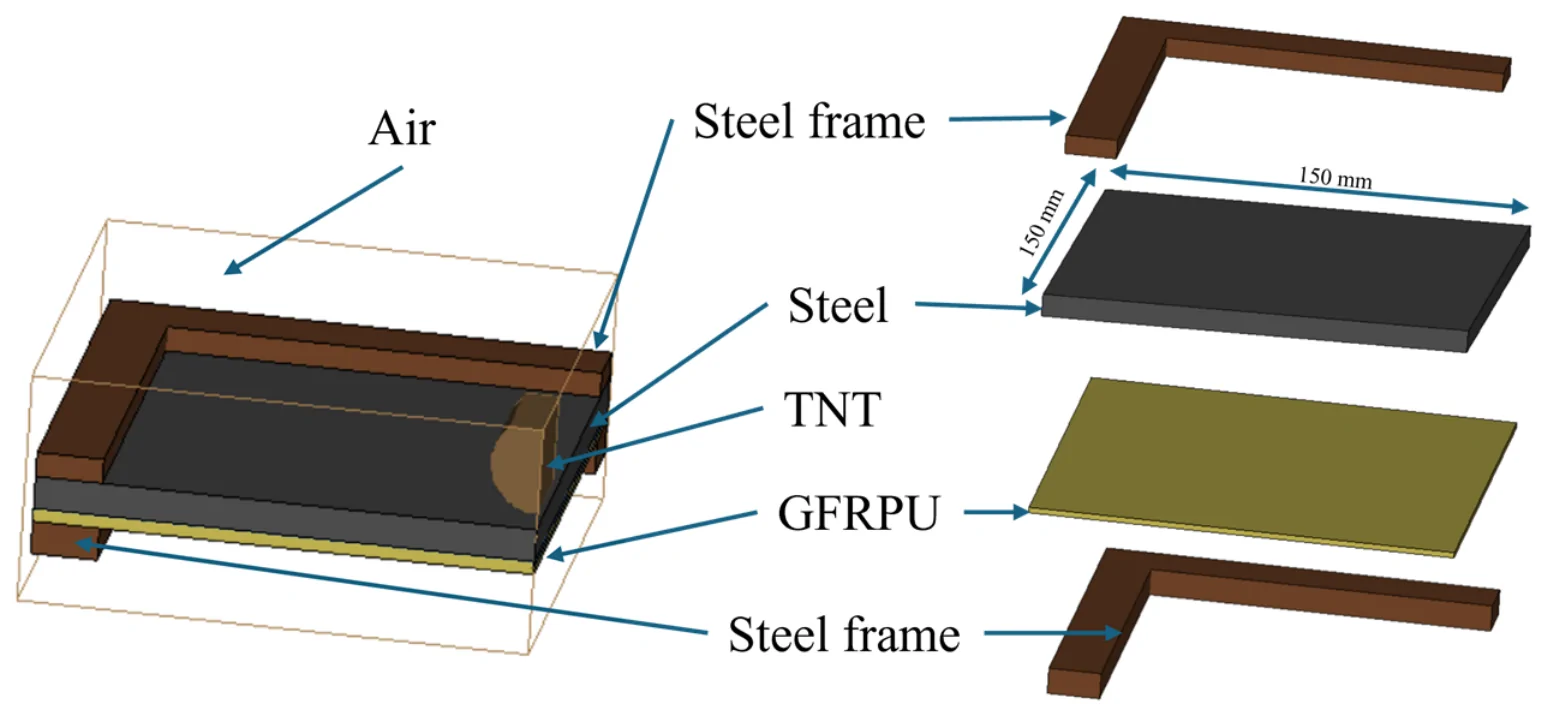
Diagram of finite element model.

A three-dimensional finite element model dimensionally consistent with the blast tests was established in ANSYS R12.2.2/LS-DYNA using the g-mm–ms–MPa unit system. The model comprises five parts: the air domain, the TNT charge, the Q235A steel plate, the GFRPU coating, and the restraining steel frame (Figure 10). The steel plate measures 300 mm × 300 mm × 8 mm, and the GFRPU coating is bonded to the back-blast face over the same in-plane extent with a thickness of 4 mm in the baseline model. The restraining frame has an inner opening of 150 mm × 150 mm, and the plate is rigidly clamped along the annular region covered by the frame; this clamped region defines the reference plane for all displacement measurements. The air domain is a 600 mm × 600 mm × 600 mm cube, and the 75 g cylindrical TNT charge (diameter 40 mm, height 45 mm) is positioned concentrically with the plate center and in direct contact with the front face. Non-reflecting boundary conditions are applied to all external air boundaries.

The air and explosive were modelled with ALE multi-material solid elements, whereas the GFRPU coating, steel plate, and steel frame were modelled with Lagrangian 8-node constant-stress solid elements. A single integration point was used through the element thickness. The Lagrangian mesh uses a characteristic element size of 3.0 mm in the refined central 150 mm × 150 mm region and coarsens gradually to 5.0 mm toward the plate edges. The GFRPU coating uses the same in-plane mesh as the adjacent steel surface and four element layers through the coating thickness. The air domain and TNT charge use ALE element sizes of 5 mm. The total element count is 1,098,000 (approximately 90,000 Lagrangian elements for the steel plate, coating and frame, and 1,008,000 ALE elements for the air domain). The detonation is initiated at the geometric center of the cylindrical TNT charge using *INITIAL_DETONATION. With the lower face of the charge in contact with the plate front face, the initiation point is located 22.5 mm above the plate front face along the charge axis.

The fluid–structure interaction between the ALE air/detonation products and the La-grangian structures is realized with *CONSTRAINED_LAGRANGE_IN_SOLID using the default penalty coupling parameters (NQUAD = 2, CTYPE = 4). The nodes of the steel plate within the clamped annular region are fully constrained in all six degrees of freedom, and the base of the steel frame is fixed. The steel–GFRPU interface is represented by *CONTACT_AUTOMATIC_SURFACE_TO_SURFACE_TIEBREAK with OPTION = 2 and the normal and shear failure strengths reported in Table 3. The default stiffness hourglass control (IHQ = 4) is employed, and the hourglass energy remained below 4.6% of the internal energy throughout the analysis. The termination time is 600 μs, and the time-step size is controlled automatically by the Courant condition with a scale factor of 0.6.

The shell element formulation is not used; all structural parts are solid. The coating–steel contact is defined as a surface-to-surface tiebreak with a segment-based penalty formulation. No separate solid layer is used for the primer, and its mechanical function is represented by the effective interface law. The complete set of LS-DYNA keyword definitions and the material card parameters are described in Section 5.2.

The full three-dimensional configuration consists of the air domain (600 × 600 × 600 mm), the Q235A steel plate (300 × 300 × 8 mm), the 4 mm GFRPU coating on the back-blast face, the restraining steel frame with a 150 × 150 mm central opening, and the 75 g cylindrical TNT charge (Φ40 × 45 mm) in contact with the front face. The 150 mm dimension denotes the central opening of the restraining frame, not a symmetry plane. The clamped annular region of the steel plate and the non-reflecting air boundaries are also indicated.

A three-level mesh-sensitivity study was conducted with Lagrangian element sizes of 4.0, 3.0 and 2.0 mm in the refined region, while the ALE element sizes were kept unchanged. For ease of comparison, only the permanent center-point deflection of the bare steel plate is reported here, which was 17.2 mm, 18.6 mm and 19.2 mm, respectively. Refining the mesh from 3.0 mm to 2.0 mm changed the permanent center-point deflection by less than 2.8%. The hourglass energy remained below 5% of the internal energy for all three meshes. On this basis and considering the computational cost, the 3.0 mm Lagrangian mesh and the 5 mm ALE mesh were adopted for all simulations.

The epoxy-modified polyurethane primer applied experimentally (Section 3.3) is approximately 50 μm thick and is not discretized as a separate solid layer. Instead, the combined mechanical response of the primer and the two adjacent bonding interfaces is represented by an effective tiebreak contact law. The normal and shear failure strengths (NFLS and SFLS) reported in Table 3 therefore represent the effective strength of the complete steel–primer–GFRPU bonding system, not the intrinsic strength of a single interface. A sensitivity study was performed in which NFLS and SFLS were varied simultaneously over the range of 1–3 MPa (i.e., ±50% around the calibrated value of 2 MPa).

### 5.2. Material Model

The *MAT_PLASTIC_KINEMATIC material model was employed for the steel plate in the model, with material parameters (Table 4) adopted from Reference [23]. The Cowper–Symonds strain-rate parameters for the polyurea matrix were taken as C = 40 s^−1^ and P = 5, based on fitting of the measured rate-dependent elastic modulus and consistent with the mild-steel values adopted for Q235A. Because the available tensile experiments cover only 0.01–100 s^−1^, the material response at local strain rates exceeding 100 s^−1^ is extrapolated by logarithmic linear extension of the highest-rate curve. It is acknowledged that the model has been calibrated only against uniaxial tensile data, and the compressive and multiaxial responses under contact explosion are not independently calibrated.

The GFRPU (density *ρ* = 1.01 g/cm^3^, Elastic modulus *E* = 96.78 MPa, Poisson’s ratio *v* = 0.4) was model by *MAT_PLASTICITY_POLYMER [16,23]. The compressive and tensile engineering stress–strain curves at different strain rates are shown in Figure 3 and Figure 4.

The equation of state for air was defined using the *EOS_LINEAR_POLYNOMIAL keyword. The explosive was TNT, for which the constitutive model was described by *MAT_HIGH_EXPLOSIVE_BURN and the equation of state by *EOS_JWL. The JWL equation of state is expressed mathematically in Equation (3), with the associated material parameters for TNT summarized in Table 5.

(3) $$p=A\left(1-\frac{\omega }{R_{1}V}\right)e^{-R_{1}V}+B\left(1-\frac{\omega }{R_{2}V}\right)e^{-R_{2}V}+\frac{\mathrm{\omega E}}{V}$$

where *p* denotes the pressure of the detonation products, *V* denotes the relative volume, and *A*, *B, R*_1_, *R*_2_ and *ω* are constants.

### 5.3. Structural Deformation Verification

The numerical model was assessed against two experimentally tested configurations under a single loading condition (75 g TNT contact explosion): a bare steel plate and a steel plate with a 4 mm back-face GFRPU coating. The bare-plate case provides an independent check of the blast-loading and steel constitutive components of the model, because the steel parameters were adopted from a cited source and not calibrated against the present bare-plate test. The 4 mm coated-plate case provides a combined calibration/verification case: the GFRPU material model was calibrated from independent uniaxial tensile tests, whereas the steel–GFRPU interface strength was calibrated against the observed debonded-zone diameter. Accordingly, the model is described as calibrated and partially verified for the two tested configurations under the specific 75 g TNT contact-explosion condition, rather than as generally validated. Predictions for untested coating thicknesses (Section 5.4) are explicitly identified as preliminary numerical results.

The quantitative comparison between experiment and simulation is limited to the permanent center-point displacement (Table 6), the approximate debonded-zone extent, the crack locations on the bare plate, and the overall deformation mode. Full-field residual deformation profiles could not be reconstructed retrospectively because only the center-point displacement and post-blast photographs were recorded.

In addition to the deformation comparison, the damage pattern of the uncoated steel plate was examined. The *MAT_PLASTIC_KINEMATIC model of the steel plate incorporates a plastic-strain-based failure criterion (effective plastic strain at failure of 0.28 [3,4,23]), so that crack initiation is represented by the erosion of elements reaching the failure strain. For the uncoated plate, the simulation predicts a pronounced concentration of effective plastic strain—and associated element erosion—around the edge of the front-face pit and at the center of the back-blast face, which coincides well with the crack locations observed in the blast test (Figure 7a,b). The model therefore captures the crack initiation behavior of the uncoated plate. It should be noted, however, that the erosion-based approach cannot explicitly resolve crack opening and propagation, and the predicted crack extent is inherently dependent on the element size; the crack morphology in the simulation is thus indicative rather than quantitatively exact.

It should be emphasized that the stress-based tiebreak law captures the initiation and approximate spatial extent of adhesive separation, but it does not reproduce progressive, energy-controlled interface degradation with the fidelity of a cohesive-zone model. Specifically, the following aspects are represented only approximately: (i) the debonding front is sharper than the experimentally observed gradual separation; (ii) the post-failure frictional sliding between the debonded coating and the steel substrate is modelled only through the residual contact penalty, without an explicit friction coefficient; (iii) the irregular, non-circular debonding front observed in the blast test (Figure 8b) is idealized as an approximately circular zone in the simulation. Because the observed debonded-zone diameter of ~100 mm was used as the calibration target for NFLS and SFLS, agreement in this quantity is treated as a calibration result and not as independent validation. The simulated debonded-zone diameter is approximately 100 mm, which matches the experimental value because this quantity was used as the calibration target for NFLS and SFLS. Agreement in this quantity is therefore treated as a calibration result rather than as independent validation. All claims regarding the “accuracy” of the interface model have been moderated accordingly. In summary, the numerical model reproduces the crack initiation locations and the overall damage pattern of the uncoated steel plate, but it does not explicitly resolve crack opening and propagation. The predicted crack extent is mesh-dependent and should be regarded as indicative rather than quantitatively exact.

### 5.4. Structural Deformation Analysis

**Figure 11:**
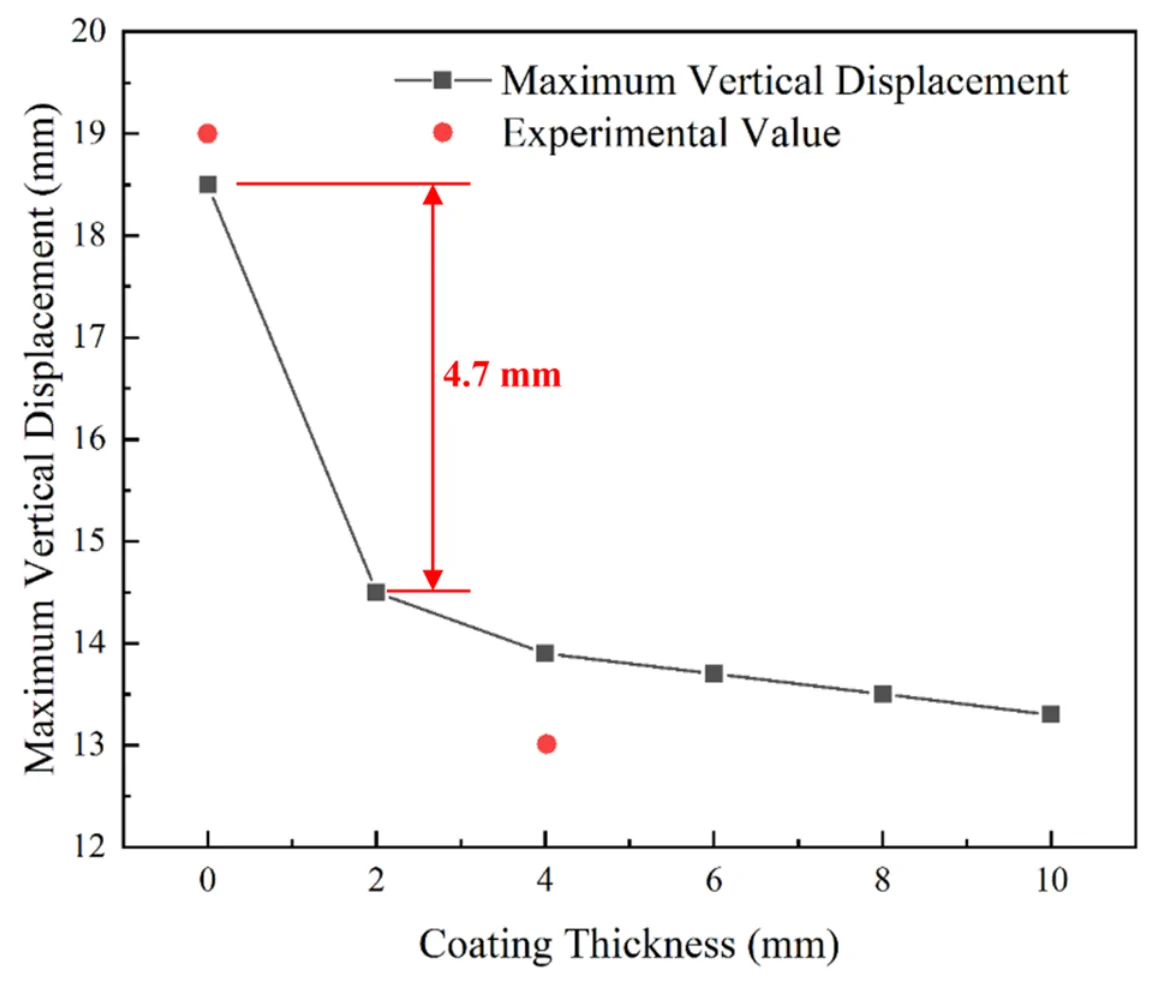
Maximum vertical displacement of steel plates coated with GFRPU of varying thicknesses.

Based on the finite element model established in this study, a preliminary investigation was conducted into the protective performance of steel plates afforded by GFRPU coatings of different thicknesses, with the aim of identifying the optimal coating thickness. The post-blast center-point maximum vertical displacements of the coated steel plates are presented in Figure 11 as a function of GFRPU coating thickness.

As shown in Figure 11, compared with the uncoated steel plate, the maximum vertical displacement of the steel plate is reduced by 4.7 mm with GFRPU protection, corresponding to a 25% decrease in deformation. However, with further increases in coating thickness, the steel plate deformation continues to decrease. The magnitude of this reduction is marginal relative to that achieved by the preceding thickness. Compared with the 2 mm GFRPU-coated steel plate, increasing the coating thickness to 4 mm reduced the maximum vertical displacement from 14.5 mm to 13.9 mm, a decrease of merely 4.14%. Here, the protective efficiency *η* is defined as:

*η* = (*δ*_0_ − *δ_t_*)/*δ*_0_ × 100% (4)

where *δ*_0_ is the maximum center-point vertical displacement of the uncoated steel plate and *δ_t_* is that of the steel plate coated with a GFRPU layer of thickness (*t*). To compare coatings of different thicknesses on a fair basis, the protective efficiency per unit thickness ( $\bar{\eta }$ ) is further defined as:

(5) $$\bar{\eta }\;=\;\eta /t$$

**Figure 12:**
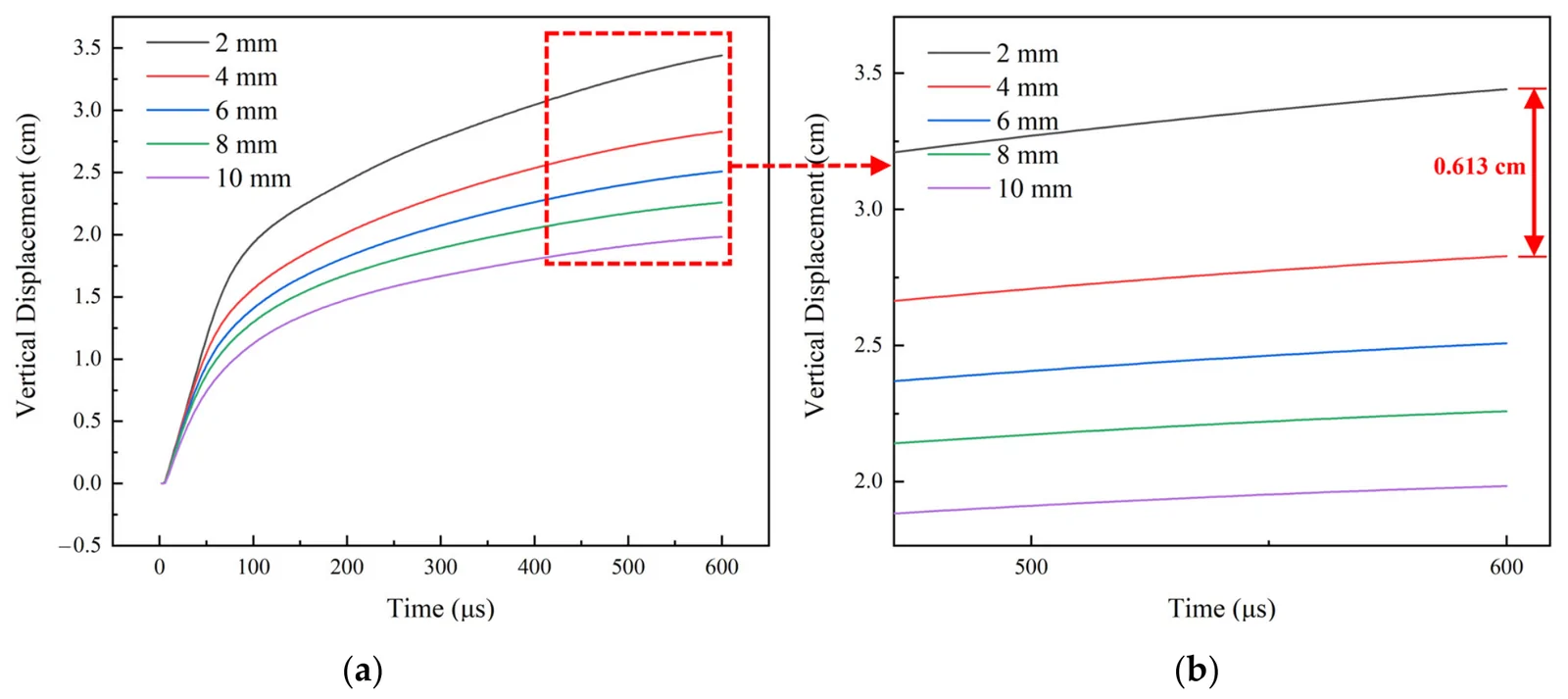
Vertical displacement of GFRPU coatings of different thicknesses: (**a**) 0~600 μs; (**b**) 500~600 μs.

With the simulated *δ*_0_ = 18.6 mm, the 2 mm GFRPU coating yields *η* = 22.0% and $\bar{\eta }$ = 11.0%/mm, whereas the 4 mm coating yields *η* = 25.3% and $\bar{\eta }$ = 6.32%/mm. In terms of protective efficiency per unit thickness, the 2 mm GFRPU coating therefore exhibits the highest protective efficiency. It should be emphasized, however, that the protective efficiency defined above accounts only for the deformation of the protected steel plate and does not reflect the integrity of the coating itself. As shown in Figure 12, the 2 mm coating undergoes a maximum displacement of 34.4 mm and loses its integrity through extensive debonding, so the 2 mm solution is efficient only in this narrow sense.

To investigate the dynamic mechanical response of GFRPU coatings subjected to blast loading, we analyzed the time-history curves of vertical displacement for coatings with varying thicknesses during the loading phase (Figure 12). Overall, the vertical displacement of GFRPU under blast loading increases initially and subsequently plateaus. This deformation behavior is consistent with the stress–strain characteristics of GFRPU. In fact, at the instant of blast loading, the response is dominated by the linear elastic deformation stage (Stage 1) of GFRPU. Thereafter, as deformation progressively increases, the plastic characteristics of the GFRPU coating assume the primary protective role.

**Figure 13:**
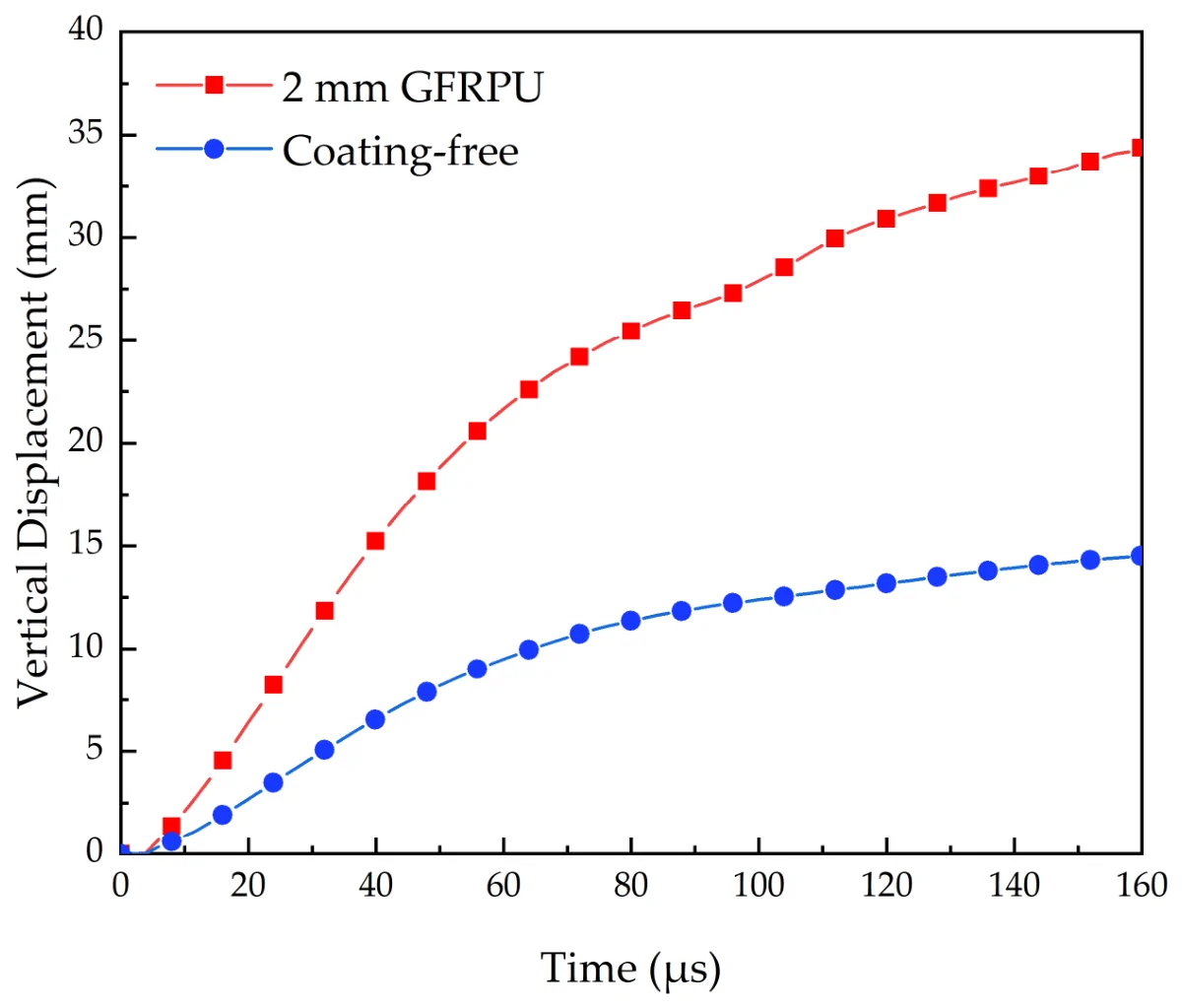
Simulated deformed configuration of the 2 mm GFRPU-coated steel plate, highlighting the measurement points of the maximum vertical displacement of the steel plate center (14.5 mm) and of the center of the coating free (back) surface (34.4 mm), both referenced to the initial undeformed configuration.

For the 2 mm-GFRPU coated steel plate, the maximum vertical displacement of the steel plate, evaluated at the center point of the plate, is 14.5 mm, whereas the maximum vertical displacement evaluated at the center of the GFRPU coating reaches 34.4 mm (Figure 13). The two values refer to different material points: once interfacial debonding occurs, the coating detaches from the steel plate and undergoes additional large deformation that is no longer constrained by the plate, so that the displacement of the coating free surface markedly exceeds that of the underlying steel plate. The large difference between the two displacements therefore indicates that adhesive failure has occurred between the GFRPU coating and the steel plate, which is consistent with the blast test results. The tensile action of the blast-induced stress wave at the structural interface exerts a pronounced influence on the integrity of the composite structure. Consistent with the blast test observations (Figure 8b), blast-induced interfacial debonding of the GFRPU coating is identified as a primary failure mechanism contributing to coating debonding from the steel substrate surface. Simultaneously, based on the microscopic damage characteristics of the GFRPU fracture surface, tensile failure of the coating itself also constitutes a significant factor. Regarding coating thickness, the vertical displacement of the coating under blast loading gradually decreases with increasing coating thickness. Specifically, when the coating thickness is increased to 4 mm, the vertical displacement of the coating decreases markedly by 6.13 mm, indicating that the increased coating thickness enhances the restraining capability of the coating. However, this enhancement is limited; when the thickness is further increased to 6 mm, the subsequent decrease in vertical displacement tends to plateau.

The optimal coating thickness is therefore determined by a combined criterion of (i) the residual deformation of the protected steel plate, (ii) the deformation and integrity of the coating itself, and (iii) the marginal benefit of additional thickness. Compared with the 2 mm coating, the 4 mm coating reduces the maximum displacement of the steel plate from 14.5 mm to 13.9 mm and, more importantly, reduces the maximum displacement of the coating from 34.4 mm to 28.27 mm (a decrease of approximately 18%), indicating that the post-debonding deformation of the coating is strongly suppressed and that its integrity is much better preserved. Increasing the thickness to 6 mm yields only marginal further reductions in both displacements, i.e., the protective benefit tends to plateau. Considering the vertical displacements of both the steel plate and the coating, the integrity of the coating itself, and the overall structural safety, the 4 mm GFRPU coating provides the most balanced protective efficiency and safety and is therefore recommended as the optimal thickness. It should nevertheless be noted that the stress-based tiebreak criterion captures the onset of debonding but does not describe progressive, energy-based interface degradation as a cohesive-zone model would.

## 6. Conclusions

Based on modified glass fiber powder, GFRPU was successfully synthesized by the spray process in this study. The mechanical properties of the polyurea and GFRPU were evaluated at varying strain rates. FTIR was used to examine the effect of modified glass fiber powder on the molecular structure of the polyurea matrix. The damage behavior of GFRPU-coated steel plates under contact explosion was investigated via 75 g TNT blast tests. Furthermore, the influence of coating thickness on the blast resistance of steel plates was also discussed. The main conclusions can be drawn as follows:

(1) Both the polyurea matrix and GFRPU are highly sensitive to strain rate. At low strain rates, the chemical crosslinks formed between the modified fibers and the matrix restrict the segmental motion of molecular chains, so the strain-rate sensitivity of GFRPU becomes weaker. At 100 s^−1^, the fibers play a major reinforcing role, raising the elastic modulus, yield stress and tensile strength of GFRPU by 13%, 24% and 26% relative to the matrix, respectively. The step-like fracture surface, on which some fibers remain embedded after pull-out, shows that the modified fibers bond well with the matrix.
(2) FTIR analysis reveals the molecular-scale origin of this reinforcement: addition of the modified glass fiber powder shortens the N–H hydrogen bonds and increases both the total hydrogen-bonding degree and the ordered hydrogen-bonded carbonyl content in the urea carbonyl region. The resulting stronger and more ordered hydrogen-bonded network accounts for the improved macroscale mechanical strength of GFRPU.
(3) Under 75 g TNT contact explosion, the 4 mm coating sprayed on the back face reduces the center deflection of the steel plate by 31.6%, and the protected plate remains free of cracks, whereas the unprotected one cracks on the back face and is close to failure. The damage of the coated plate is mainly governed by interface debonding under the blast-induced tensile stress wave, and the glass transition of polyurea at high loading rates further promotes brittle fracture of the coating. It is worth noting that the fibers embedded in the coating redistribute the local stress and thus hinder brittle cracking of the nearby matrix.
(4) The simulated center displacements match the test data well, with errors of only −2.1% and 6.9%. A 2 mm coating gives the highest protective efficiency per unit thickness, but its large free-surface displacement indicates extensive debonding and loss of coating integrity. Increasing the thickness to 4 mm markedly suppresses the post-debonding deformation of the coating and further reduces the plate deflection, whereas further thickening brings little extra benefit. On balance, 4 mm is recommended as the optimal thickness, taking account of plate deformation, coating integrity and overall structural safety.

From a practical standpoint, by retaining the one-step spray-processing route of conventional polyurea while simultaneously improving the mechanical properties of the coating, altering its mechanical behavior and fracture mechanism, and enhancing its damage tolerance under blast loading, GFRPU is a promising candidate for the blast hardening of steel structures, since existing structures can be retrofitted on their protected back faces without modifying the exposed surfaces. Several limitations of this work should be emphasized. First, the simulated GFRPU was calibrated from the as-prepared composite, thus excluding fiber–matrix interfacial adhesion and stress wave propagation. Second, the blast validation is limited to two configurations under a single charge mass, with one specimen tested per configuration. Finally, the long-term environmental durability of GFRPU remains to be established: although our preliminary study [10] examined environmental effects on polyurea coatings for water-proofing, GFRPU is a heterogeneous composite whose durability under complex blast-mitigation conditions depends on coupled aging mechanisms and stringent stability requirements. Future work will therefore implement a systematic aging protocol—UV, wet-heat, and salt spray exposure followed by quantitative assessment of residual tensile strength, elongation at break, and blast resistance.

## Figures and Tables

**Table 1:** Main components.

Components	Size (Mesh)	Manufacturer
Glass fiber powder	100	Jiangsu Ruixin mining Co., Ltd., Changzhou, China
3-(azidopropyl)triethoxysilane (KH550)	-	China National Pharmaceutical Group Corporation, Beijing, China
Shamu T4 polyurea component A	-	Qingdao Shamu Advanced Material Co., Ltd., Qingdao, China
Shamu T4 polyurea component B	-	Qingdao Shamu Advanced Material Co., Ltd., Qingdao, China

**Table 2:** Carbonyl stretching region absorption peak attribution and percentage of hydrogen bonding in Shamu T4 and GFRPU.

Type	Wavenumber /cm^−1^	Assignment	Area	Percentage of Hydrogen Bonding (%)		
				X_o_	X_diso_	X_b_
Shamu T4	1583–1611	Ordered hydrogen bonding	2.217	56.8		80.0
	1635–1658	Disordered hydrogen bonding	0.903		23.2	
	1703–1718	Free	0.780			
GFRPU	1587–1617	Ordered hydrogen bonding	4.591	59.2		84.1
	1635–1654	Disordered hydrogen bonding	1.935		24.9	
	1693–1718	Free	1.229			

Note: X_o_ and X_diso_ are the percentages of ordered hydrogen bonding and disordered hydrogen bonding. X_b_ is the degree of hydrogen bonding.

**Table 3:** Main parameter of contact automatic tiebreak.

OPTION	NFLS (GPa)	SFLS (GPa)	PARAM	ERATEN	ERATES	CT2CN	CN
1	1.51 × 10^−4^	2.71 × 10^−4^	1	0	0	1	0

**Table 4:** Material parameters of Q235A steel plate [23].

Density *ρ* (kg·m^−3^)	Elastic Modulus *E* (GPa)	Poisson’s Ratio *ν*	Yield Stress *σ* (MPa)	Tangent Modulus *E*_tan_ (MPa)	Cowper–Symonds C (s^−1^)	Cowper–Symonds P	Effective Plastic Strain at Failure
7850	210	0.3	235	6100	40	5	0.28

**Table 5:** Material parameters of TNT.

*ρ* (g·cm^−3^)	*D* (m·s^−1^)	*P*_CJ_ (GPa)	*A* (GPa)	*B* (GPa)	*R*_1_	*R*_2_	*ω*	*E*_0_ (GPa)	*V*_0_
1.58	6880	19.4	307	3.898	4.15	0.95	0.30	7.0	1.0

Note: *D* is detonation velocity of TNT. *P*_CJ_ is Chapman–Jouguet pressure.

**Table 6:** Comparison of final vertical displacement at central point between experimental results and numerical simulations.

Type	Experimental Results (mm)	Numerical Simulations (mm)	Inaccuracies (%)	Status of Comparison
Steel plate	19.0 ± 0.2	18.6	−2.1	Independent check
GFRPU coated steel plate	13.0 ± 0.1	13.9	6.9	Calibration/verification

## Data Availability

The original contributions presented in this study are included in the article. Further inquiries can be directed to the corresponding authors.

## Abbreviations

The following abbreviations are used in this manuscript:

GFRP	glass fiber-reinforced polymer
GFRPU	glass fiber-reinforced polyurea
FTIR	Fourier transform infrared spectroscopy
SEM	scanning electron microscope
ALE	Arbitrary Lagrangian-Eulerian
